# Efficacy of combination of Ezetimibe 10 mg and rosuvastatin 2.5 mg versus rosuvastatin 5 mg monotherapy for hypercholesterolemia in patients with type 2 diabetes

**DOI:** 10.1186/1476-511X-12-137

**Published:** 2013-09-22

**Authors:** Keiichi Torimoto, Yosuke Okada, Hiroko Mori, Maiko Hajime, Kenichi Tanaka, Akira Kurozumi, Manabu Narisawa, Sunao Yamamoto, Tadashi Arao, Hirofumi Matsuoka, Nobuo Inokuchi, Yoshiya Tanaka

**Affiliations:** 1 First Department of Internal Medicine, School of Medicine, University of Occupational and Environmental Health, 1-1 Iseigaoka, Yahatanishi-ku, Kitakyushyu-shi 807-8555, Japan; 2 Matsuoka Clinic, Fukuoka, Japan; 3 Inokuchi Clinic, Fukuoka, Japan

**Keywords:** Rosuvastatin, Ezetimibe, Hypercholesterolemia, Type 2 diabetes mellitus

## Abstract

**Background:**

Statins are used to treat hypercholesterolemia in patients with type 2 diabetes mellitus, but many of these patients fail to achieve the target LDL-C level. Recent reports have suggested that a synergistic effect can be obtained by concomitant administration of the cholesterol absorption inhibitor ezetimibe and a statin. However, in patients with type 2 diabetes who are already being treated with satins, it remains unclear whether it is more effective to add ezetimibe or to increase the statin dose. Therefore, this study was performed to examine the effects of these two regimens on LDL-C and lipoproteins.

**Methods:**

The subjects were type 2 diabetic patients under treatment with rosuvastatin (2.5 mg daily), who had LDL-C levels ≥80 mg/dL. They were randomly allocated to a group that received add-on therapy with ezetimibe at 10 mg/day (combination group, n = 40) or an increase of the rosuvastatin dose to 5 mg/day (dose escalation group, n = 39). These two groups were compared at baseline and after 12 weeks of treatment.

**Results:**

The percent change of LDL-C was −31% in the combination group and −12% in the dose escalation group. Both groups showed a significant decrease, but the decrease was greater in the combination group. In both groups, there was a significant decrease in the levels of small dense LDL-C, oxidized LDL and remnant-like lipoprotein cholesterol. For all of these parameters, the percent changes were greater in the combination group. Only the combination group showed a significant decrease of triglycerides. Multivariate analysis was performed to identify factors associated with reaching an LDL-C level <80 mg/dL. As a result, add-on therapy with ezetimibe was extracted as a factor related to improvement of LDL-C.

**Conclusions:**

Compared with increasing the dose of rosuvastatin, the combination of rosuvastatin and ezetimibe not only achieves quantitative but also qualitative improvement of serum lipid levels in type 2 diabetic patients, suggesting that this combination could suppress the progression of atherosclerosis.

**Trial registration:**

UMIN000011005

## Background

Type 2 diabetes mellitus is an important risk factor for atherosclerotic diseases. A meta-analysis has shown that the risk of developing coronary artery disease and stroke increased by 2.0-fold and 2.3-fold, respectively, in patients with diabetes mellitus [1]. A recent report on the Japan Diabetes Complications Study (JDCS), which is being conducted in patients with type 2 diabetes, showed that both LDL-C and TG are risk factors for cardiovascular events [2], and improvement of dyslipidemia in patients with diabetes is believed to contribute greatly to preventing the development of atherosclerosis.

Characteristics of lipid metabolism in patients with diabetes include susceptibility to development of hyper-LDL-cholesterolemia, hypertriglyceridemia, and hypo-HDL-cholesterolemia, in addition to quantitative and qualitative abnormalities of lipoproteins, such as an increase of small dense LDL-cholesterol (sdLDL-C), oxidized cholesterol (oxidized low-density lipoprotein cholesterol; oxidized LDL), and remnant lipoprotein [3]. Although many patients with type 2 diabetes currently receive treatment based around statins, the achievement rate of the target LDL-C level remains low with statin therapy alone [4].

Ezetimibe inhibits Niemann-Pick C1-like protein 1 (NPC1L1), a cholesterol transporter that exists in the small intestinal mucosa, and reduces serum cholesterol by suppressing the absorption of dietary and biliary cholesterol from the small bowel [5]. In recent years, concomitant use of statins and ezetimibe has been reported to have a greater LDL-lowering effect [6]. For example, the SHARP study showed that LDL-C-lowering therapy using the combination of ezetimibe and a statin led to a decrease of atherosclerotic events [7]. However, only a few studies have directly compared whether it is better to increase the statin dose or to add ezetimibe to basal statin therapy in hypercholesterolemic patients with type 2 diabetes who have not shown a sufficient response to statin monotherapy.

The present study investigated hypercholesterolemic patients with type 2 diabetes on rosuvastatin at 2.5 mg/day, whose levels of LDL-C levels were higher than 80 mg/dL despite this treatment. These patients were randomly allocated to a group that received a higher dose of rosuvastatin (5 mg/day) or a group that received add-on therapy with ezetimibe at 10 mg/day. The effects on LDL-C and on qualitative improvement of atherosclerosis-inducing lipoproteins were compared after 12 weeks of administration.

## Methods

### Subjects

The subjects of this study (UMIN000011005) were hypercholesterolemic patients with type 2 diabetes aged from 20 years to less than 80 years, who had been receiving rosuvastatin (2.5 mg/day) for more than 12 weeks but had LDL-C levels higher than 80 mg/dL, whose therapeutic regimen had not been changed for the past three months, and who had an HbA1c (NGSP) of less than 8.4%. In the JAPAN-ACS Study [8] and the COSMOS Study [9], progression of coronary plaque was significantly prevented by reducing the LDL-C level to 75 mg/dL and 80 mg/dL, respectively. Also in the JART Study [10], progression of coronary plaque was significantly suppressed in the consolidation therapy group (mean LDL-C level: 83.7 mg/dL) compared with the standard therapy group (mean LDL-C level: 117.4 mg/dl). In a cohort study of Japanese diabetic patients without a history of cardiovascular disease, the incidence of cardiovascular events was higher among the patients with LDL-C levels above 80 mg/dL [11]. Based on these reports, we selected diabetic patients with LDL-C levels higher than 80 mg/dL for the present study. Patients with a history of familial hypercholesterolemia, patients who had developed stroke or ischemic heart disease within the past six months, patients with liver failure (ALT and/or AST >80 IU/L) or kidney failure (serum creatinine >1.3 mg/dL), patients using insulin, patients who were pregnant or could possibly become pregnant, and patients who were breast feeding were excluded from the study. All patients were Japanese. This study was conducted with the approval of the Ethics Committee of the University of Occupational and Environmental Health, Japan, and written consent to the study was obtained from all subjects.

### Study protocol

The study employed a randomized open-label design. Patients with type 2 diabetes on treatment with rosuvastatin at 2.5 mg/day (n = 79) were randomly allocated to two groups by the envelope method: the add-on ezetimibe group (combination group) that received 2.5 mg/day of rosuvastatin and 10 mg/day of ezetimibe (n = 40), and the rosuvastatin dose escalation group that received 5 mg/day of rosuvastatin (n = 39). During the study period, patients were prohibited from receiving new drugs or discontinuing drugs, changing the dosage and administration, or undergoing new lifestyle modification including exercise therapy and diet therapy. Evaluation was performed at the beginning of the study and 12 weeks after initiation of the study by fasting blood tests. The study endpoints were serum lipids, including LDL-C, HDL-C, sdLDL-C, oxidized LDL (malondialdehyde-modified low-density lipoprotein; MDA-LDL), TG, and remnant-like particle cholesterol (RLP-C), as well as other clinical tests such as fasting blood glucose, HbA1c, fasting serum insulin, and high sensitivity C-reactive protein. These were measured at baseline and after 12 weeks.

The primary endpoint was set as the percent change of LDL-C after 12 weeks compared with baseline. Secondary endpoints were the percent changes of HDL-C, sdLDL-C, oxidized LDL (MDA-LDL), TG, and RLP-C after 12 weeks compared with baseline.

### Laboratory tests

Venous blood sampling was performed early in the morning after fasting for over 12 hours. Serum lipids were measured using the Hitachi 7350 autoanalyzer (Hitachi Co., Tokyo, Japan). LDL-C, HDL-C, and TG were measured by enzymatic methods, and LDL-C was measured by the direct method. sdLDL-C was measured using the sdLDL-EX reagent “SEIKEN” (Denka Seiken Inc., Tokyo, Japan) and a Hitachi 7170 autoanalyzer (Hitachi Co., Tokyo, Japan) by the homogeneous assay [12]. MDA-LDL was measured by a sandwich ELISA method using the oxidative ELISA “Daiichi” (Sekisui Medical, Tokyo, Japan). RLP-C was measured using the RLP-C reagent “JIRO-II” (Otsuka Inc., Tokyo, Japan) and the Hitachi 7170 autoanalyzer (Hitachi Co., Tokyo, Japan). The insulin resistance index (HOMA-IR) was calculated by the following formula: fasting IRI (mU/L) × fasting blood glucose (mg/dL)/405. HbA1c (%) was measured by HLPC with a Tosoh HLC-723 G8 (Tosoh Co., Kyoto, Japan), and results were expressed as National Glycohemoglobin Standardization Program (NGSP) values by adding 0.4% to the conventional Japanese standard substance (JDS) HbA1c values (JDS values) [13].

### Statistical analysis

Results are expressed as the mean ± standard deviation. Assuming that the LDL-C lowering effect was 21% when rosuvastatin and ezetimibe were used concomitantly, and 6% when the rosuvastatin dose was doubled [14], the number of subjects required was calculated to be 32 patients per group according to the t-test (two-tailed hypothesis) with the significance level α and power 1-β set as 5% and 90%, respectively. Assuming a dropout rate of 20%, the target sample size of 80 patients was established. Normality of data distributions was assessed using the Shapiro-Wilk test. Comparison of baseline data and 12-week data was performed using the paired t-test if a normal distribution was found, or the Wilcoxon signed rank test was used when the distribution was not normal. Comparison of percent changes between the two groups was performed using Student’s t-test if a normal distribution was found, or the Mann–Whitney U-test was used if the distribution was not normal. For categorical data, Fisher’s exact probability test was employed when the expected cell size was less than 5, while the chi-square test was used otherwise. Univariate and multivariate logistic regression analyses were performed by classifying patients with LDL-C <80 mg/dL at Week 12 as the improved group, and others as the unimproved group. The odds ratio and 95% confidence interval are shown for all data. Multivariate logistic regression analysis was performed using step-up procedures with factors showing p < 0.25 in univariate logistic regression analysis, from which factors showing multicollinearity based on the Spearmann’s rank correlation analysis were removed. Analyses were performed using SPSS Statistical Software 21.0 (SPSS Inc., Chicago, IL), and P < 0.05 was considered significant.

## Results

### Baseline clinical characteristics of the subjects

The subjects were 79 patients (men, 41; women, 38), including 40 patients in the add-on ezetimibe group (combination group) that received 2.5 mg/day of rosuvastatin and 10 mg/day of ezetimibe, and 39 patients in the increased rosuvastatin dose group (dose escalation group) that received 5 mg/day of rosuvastatin. Of these, one patient in the combination group and three patients in the dose escalation group dropped out due to discontinued hospital attendance. Consequently, analysis was performed on 39 patients in the combination group and 36 patients in the dose escalation group. Age, sex, body mass index (BMI), systolic blood pressure, diastolic blood pressure, waist circumference, and the presence of metabolic syndrome (MetSyn) were comparable between the combination and dose escalation groups (Table 1).

**Table 1:** Baseline clinical characteristics of study participatns

	**Rosuva/Eze 2.5/10 mg (*n*** **= 39)**	**Rosuvastatin 5 mg (*n*** **= 36)**	***P *value**
Age, years	66.3 ± 11.7	63.0 ± 13.0	0.210
Male gender, n (%)	24 (62)	16 (44)	0.138
BMI, kg/m^2^	25.1 ± 3.6	26.5 ± 4.5	0.143
Systolic blood pressure, mmHg	127.4 ± 14.9	126.7 ± 13.8	0.849
Diastolic blood pressure, mmHg	72.2 ± 10.8	72.9 ± 8.0	0.754
Waist circumference, cm	90.5 ± 11.8	93.1 ± 11.0	0.360
MetSyn, (%)	17 (50)	18 (58)	0.515
Prevalence CVD, n (%)	4 (10)	2 (6)	0.376
Prevalence CI, n (%)	4 (10)	4 (11)	0.598
Hypertension, n (%)	28 (72)	23 (64)	0.463
Diabetes therapy			
Oral hypoglycemic agent, n (%)	30 (77)	30 (83)	0.488
sulfonylurea, n (%)	16 (41)	19 (53)	0.143
pioglitazone, n (%)	17 (44)	15 (42)	0.866
metformin, n (%)	14 (36)	15 (42)	0.608
α-glucosidase inhibitor, n (%)	8 (21)	3 (12)	0.136
dipeptidyl peptidase-4 inhibitor, n (%)	3 (8)	4 (11)	0.454
Aspartate aminotransferase, U/L	27.2 ± 12.5	21.9 ± 5.6	0.596
Alanine aminotranserase, U/L	28.2 ± 16.3	21.2 ± 9.8	0.216
Creatine kinase, IU/L	130 ± 87	118 ± 63	0.489
Creatinine, mg/dL	0.8 ± 0.3	0.7 ± 0.2	0.316

Data are mean ± SD, n, or n (%). *Rosuva* Rosuvastatin, *Eze* Ezetimibe, *BMI* Body mass index, *MetSyn* Metabolic syndrome [diagnosed by the criteria in japan], *CVD* Cardiovascular disease, *CI* Cerebral infarction.

*P* values are for differences between two groups at baseline.

### Effect on clinical parameters

**Figure 1:**
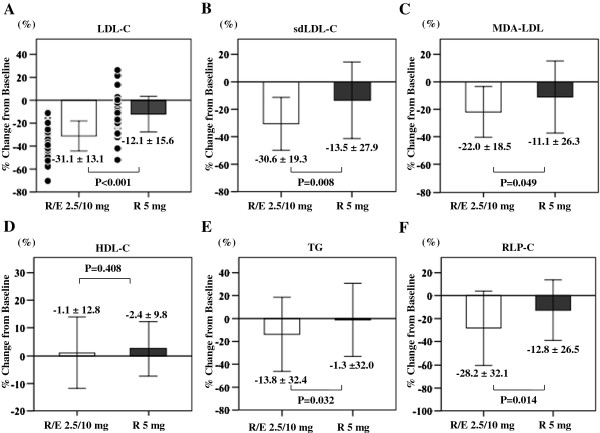
**Percent changes from baseline of Serum lipids.** Mean percent change from baseline (mean [SD]) of low-density lipoprotein cholesterol (LDL-C) **(A)**, small dense low-density lipoprotein cholesterol (sdLDL-C) **(B)**, malondialdehyde-modified low-density lipoprotein (MDA-LDL) **(C)**, high-density lipoprotein cholesterol (HDL-C) **(D)**, triglycerides (TG) **(E)**, and remnant-like particle cholesterol (RLP-C) **(F)** after 12 weeks of treatment. R/E 2.5/10 mg indicates rosuvastatin 2.5 mg + add-on ezetimibe 10 mg; R 5 mg indicates rosuvastatin 5 mg. Open columns indicate R/E 2.5/10 mg; filled columns indicate R 5 mg. Values are the means ± SD. Data were compared by Student’s t-test for normally distributed variables and the Mann–Whitney U test for variables with a skewed distribution.

Serum lipids at Week 0 and Week 12 in both groups are shown in Table 2. Percent changes from Week 0 are shown in Figure 1.

**Table 2:** Serum lipid parameters and Patients achieving specified LDL-C target at baseline and after 12 weeks of treatment

**Serum lipid parametes**	**Baseline**	**12 weeks**	^*^P value
**LDL-C, mg/dL**			
Rosuva/Eze 2.5/10 mg	111 ± 26	75 ± 18	< 0.001
Rosuva 5 mg	112 ± 22	98 ± 22	< 0.001
**sdLDL-C, mg/dL**			
Rosuva/Eze 2.5/10 mg	34 ± 11	22 ± 7	< 0.001
Rosuva 5 mg	34 ± 11	28 ± 11	0.001
**MDA-LDL, U/L**			
Rosuva/Eze 2.5/10 mg	112 ± 29	86 ± 22	< 0.001
Rosuva 5 mg	117 ± 34	100 ± 30	0.003
**HDL-C, mg/dL**			
Rosuva/Eze 2.5/10 mg	57 ± 16	57 ± 16	0.861
Rosuva 5 mg	56 ± 11	58 ± 13	0.185
**LDL/HDL ratio**			
Rosuva/Eze 2.5/10 mg	2.1 ± 0.8	1.4 ± 0.4	< 0.001
Rosuva 5 mg	2.0 ± 0.5	1.8 ± 0.5	< 0.001
**TG, mg/dL**			
Rosuva/Eze 2.5/10 mg	147 ± 71	118 ± 56	0.001
Rosuva 5 mg	147 ± 79	136 ± 68	0.700
**RLP-C, mg/dL**			
Rosuva/Eze 2.5/10 mg	4.9 ± 2.9	3.0 ± 1.1	< 0.001
Rosuva 5 mg	4.7 ± 2.5	3.7 ± 1.4	0.004
**Patients achieveing specified LDL-C target**	**Baseline**	**12 weeks**	
**Patients achieving LDL-C < 80, n (%)**			
Rosuva/Eze 2.5/10 mg	0 (0)	24 (61.5)^*†*^	
Rosuva 5 mg	0 (0)	8 (22.2)	
**Patients achieving LDL-C < 100, n (%)**			
Rosuva/Eze 2.5/10 mg	17 (43.6)	35 (89.7)^*†*^	
Rosuva 5 mg	11 (30.6)	21 (58.3)	

Data are mean ± SD, n (%). *Rosuva* Rosuvastatin, *Eze* Ezetimibe, *LDL* Low-density lipoprotein cholesterol, *sdLDL-C* Small dense low-density lipoprotein cholesterol, *MDA-LDL* Malondialdehyde-modified low-density lipoprotein, *sdLDL-C* Small dense low-density lipoprotein cholesterol, *HDL-C* High-density lipoprotein cholesterol, *TG* Triglycerides, *RLP-C* Remnant-like particle cholesterol.

There were no significant differences among each baseline values.

*P values are for differences between baseline with 12 week.

^†^P <0.01 for determine the association between-treatment difference, the Fisher’s exact test was used.

The LDL-C level, which was the primary endpoint, showed a significant decrease after 12 weeks relative to baseline in both groups. The combination group reduced the serum concentration of LDL-C from 111 ± 26 mg/dL to 75 ± 18 mg/dL (p < 0.001) and the dose escalation group from 112 ± 22 mg/dL to 98 ± 22 mg/dL (p < 0.001). Comparison between the two groups revealed that the percent change of LDL-C was −31% in the combination group and −12% in the dose escalation group, showing a significant decrease in the combination group (p < 0.001). While the LDL-C level <100 mg/dL increased from 31% to 58% in the dose escalation group, it increased from 44% to 90% in the combination group, and the increase was significantly greater in the combination group (p = 0.002). In addition, the rate of achieving an LDL-C level <80 mg/dL increased from 0% to 22% in the dose escalation group and from 0% to 62% with ezetimibe therapy, and the combination group showed significantly greater improvement (p = 0.001). The factors influencing improvement of LDL-C were examined by classifying patients with an LDL-C level <80 mg/dl in Week 12 as the improved group and others as the unimproved group (Table 3). As a result of multivariate logistic regression analysis, the treatment and the LDL-C level in Week 0 were extracted as factors affecting improvement of LDL-C: the chance of being in the LDL-C improved group was 6.29 times higher for patients from the combination group compared with the dose escalation group (95% CI 1.89-20.91), and it increased by 0.95-fold as LDL-C in Week 0 increased by 1 mg/dL (95% CI 0.92-0.98).

**Table 3:** Baseline characteristics associated with achieving LDL-C < 80 mg/dl as analyzed by univariate and multiple logistic regression analyses

	**Univariate logistic regression**			**Multiple logistic regression**		
	**Wald Χ**^**2**^	**P**	**OR (95% CI)**	**Wald Χ**^**2**^	**P**	**OR (95% CI)**
Group, Rosuva/Eze vs. Rosuva	11.0	0.001	5.60 (2.03-15.5)	9.0	0.003	6.29 (1.89-20.91)
Age, per year	0.5	0.461	1.02 (0.97-1.06)			
Male gender, male vs. female	3.3	0.068	0.42 (0.16-1.07)			
BMI, per kg/m2	0.008	0.930	1.00 (0.89-1.11)			
Systolic blood pressure, per mmHg	0.002	0.964	1.00 (0.97-1.03)			
Diatolic blood pressure, per mmHg	1.8	0.181	0.97 (0.92-1.02)			
Waist circumference, per cm	0.07	0.790	1.01 (0.96-1.05)			
MetSyn, yes vs. no	0.001	0.969	0.98 (0.37-2.63)			
Prevalence CVD, yes vs. no	0.1	0.706	1.38 (0.26-7.33)			
Prevalence CI, yes vs. no	3.3	0.069	4.73 (0.89-25.23)			
Hypertension, yes vs. no	0.01	0.904	1.06 (0.40-2.84)			
Diabetes therapy						
Oral hypoglycemic agent, yes vs. no	1.9	0.169	0.42 (0.12-1.45)			
sulfonylurea, yes vs. no	0.05	0.815	1.12 (0.43-2.90)			
pioglitazone, yes vs. no	0.1	0.758	0.86 (0.34-2.19)			
metformin, yes vs. no	1.6	0.210	1.83 (0.71-4.69)			
α-glucosidase inhibitor, yes vs. no	0.7	0.392	1.75 (0.48-6.36)			
dipeptidyl peptidase-4 inhibitor, yes vs. no	0.000	0.991	1.01 (0.21-4.86)			
LDL-C, per mg/dL	8.2	0.004	0.97 (0.94-0.99)	10.6	0.001	0.95(0.92-0.98)
sdLDL-C, per mg/dL	4.5	0.034	0.95 (0.90-1.00)			
MDA-LDL, per U/L	3.3	0.070	0.99 (0.97-1.00)			
HDL-C, per mg/dL	3.5	0.061	0.96 (0.93-1.00)			
LDL/HDL ratio	0.2	0.677	0.86 (0.43-1.74)			
TG, per mg/dL	0.4	0.526	1.00 (0.99-1.00)			
RLP-C, per mg/dL	0.2	0.637	0.96 (0.80-1.15)			
FPG, per mg/dL	0.07	0.784	1.00 (1.00-1.02)			
Fasting serum insulin, per μU/mL	0.009	0.926	1.00 (0.94-1.06)			
HOMA-IR	0.004	0.949	1.00 (0.82-1.24)			
HOMA-B	0.1	0.739	1.00 (0.99-1.00)			
HbA1c, per %	0.3	0.559	1.2 (0.69-2.00)			
Aspartate aminotransferase, per U/L	1.6	0.204	1.03 (0.98-1.08)			
Alanine aminotranserase, per U/L	3.6	0.058	1.04 (1.00-1.07)			
Creatine kinase, per mg/dL	1.03	0.311	1.00 (0.99-1.00)			
Creatine, per mg/dL	2.8	0.096	5.7 (0.73-44.58)			

Only factors with P < 0.025 on univariate logistic regressions, which were pioglitazone, LDL-C, Alanine aminotranserase, were included in this multiple factor logistic regression.

*Rosuva* Rosuvastatin, *Eze* Ezetimibe, *BMI* Body mass index, *MetSyn* Metabolic syndrome [diagnosed by the criteria in japan], *CVD* Cardiovascular disease, *CI* Cerebral infarction, *LDL* Low-density lipoprotein cholesterol, *sdLDL-C* Small dense low-density lipoprotein cholesterol, *MDA-LDL* Malondialdehyde-modified low-density lipoprotein, *HDL-C* High-density lipoprotein cholesterol, *TG* Triglycerides, *RLP-C* Remnant-like particle cholesterol, *FPG* Fasting plasma glucose, *HOMA-IR* Homeostasis model assessment of insulin resistance, *HOMA-B* HOMA of β-cell function.

sdLDL-C levels decreased significantly after 12 weeks relative to baseline in both groups. The percent change of sdLDL-C was −31% in the combination group and −14% in the dose escalation group, showing a significant decrease (p = 0.008) in the combination group.

MDA-LDL levels decreased significantly after 12 weeks relative to baseline in both groups. The percent change of MDA-LDL was −22% in the combination group and −11% in the dose escalation group, showing a significant decrease (p = 0.049) in the combination group.

HDL-C showed no significant change in both groups.

TG levels only decreased significantly in the combination group. The percent change of TG was −14% in the combination group and −1% in the dose escalation group, showing a significant decrease (p = 0.032) in the combination group.

RLP-C levels decreased significantly after 12 weeks relative to baseline in both groups. The percent change of RLP-C was −28% in the combination group and −13% in the dose escalation group, showing a significant decrease (p = 0.014) in the combination group.

Fasting blood glucose, fasting IRI, HOMA-IR, and HbA1c showed no significant differences between Week 0 and Week 12 in both groups (Table 4).

**Table 4:** Indices of glucose metabolism at baseline and after 12 weeks of treatment

	**Baseline**	**12 weeks**	***P *value**
**FPG, mg/dL**			
Rosuva/Eze 2.5/10 mg	131 ± 25	135 ± 30	0.278
Rosuva 5 mg	126 ± 21	125 ± 22	0.848
**Fasting serum insulin, μU/mL**			
Rosuva/Eze 2.5/10 mg	8.5 ± 6.9	8.3 ± 6.0	0.435
Rosuva 5 mg	9.1 ± 9.2	9.8 ± 11.0	0.128
**HOMA-IR**			
Rosuva/Eze 2.5/10 mg	2.8 ± 2.4	2.8 ± 2.4	0.983
Rosuva 5 mg	2.7 ± 2.4	3.0 ± 3.0	0.162
**HOMA-B**			
Rosuva/Eze 2.5/10 mg	48 ± 38	45 ± 26	0.412
Rosuva 5 mg	62 ± 94	68 ± 97	0.130
**HbA1c,%**			
Rosuva/Eze 2.5/10 mg	6.7 ± 0.6	6.8 ± 0.7	0.103
Rosuva 5 mg	6.9 ± 0.7	6.9 ± 0.8	0.907

Data are mean ± SD.

*Rosuva* Rosuvastatin, *Eze* Ezetimibe, *FPG* Fasting plasma glucose, *HOMA-IR* Homeostasis model assessment of insulin resistance, *HOMA-B* HOMA of β-cell function.

There were no significant differences among each baseline values.

*P* values are for differences between baseline with 12 week.

Both groups exhibited no significant increases of CK or ALT and AST (data not shown), confirming good tolerability.

## Discussion

In this study, combination therapy with ezetimibe had a stronger LDL-C lowering effect than an increased dose of rosuvastatin in patients with type 2 diabetes who maintained LDL-C levels higher than 80 mg/dL despite treatment with 2.5 mg/day of rosuvastatin. In addition, combination therapy led to further improvement of sdLDL-C, MDA-LDL, TG, and RLP-C, suggesting that the qualitative improvement of highly atherogenic lipoproteins was also greater.

Statins inhibit cholesterol synthesis in the liver, but this result in enhanced cholesterol absorption and that decreases the cholesterol-lowering effect [15]. Thus, addition of ezetimibe, a cholesterol absorption inhibitor with a complementary action to statins, had a greater LDL-C-lowering effect. In addition, the percent reduction of LDL-C was −31% in the group that received ezetimibe, which was equal to the previously reported range in patients with dyslipidemia [6,14]. Vaverkova et al. and Gianluca et al. [16,17] reported that, similar to the results of the present study, the reduction of LDL-C with combined use of ezetimibe was higher among patients with type 2 diabetes compared to those with normal glucose tolerance. Cholesterol absorption from the small intestine is facilitated by the NPC1L1 transporter. In patients with diabetes, expression of NPC1L1 is enhanced [18], whereas there is reduced expression of ATP-binding cassette transporters (ABC) G5 and G8, which are involved in returning cholesterol from small intestinal epithelial cells to the small bowel lumen [19]. Accordingly, patients with diabetes exhibit increased cholesterol absorption. Based on this background, it is considered likely that combination therapy with ezetimibe will have a more powerful effect in hypercholesterolemic patients with diabetes whose cholesterol absorption is enhanced, given that ezetimibe selectively inhibits cholesterol absorption from the intestine by binding to the NPC1L1 receptor.

In recent years, it has become clear that a decrease in the size of LDL particles is associated with the risk of coronary artery disease, and people who mainly have sdLDL-C, as opposed to those with normal-sized LDL, are known to show a three-fold higher incidence of CHD [20]. On the other hand, it has been reported that patients with type 2 diabetes often have sdLDL-C [21], suggesting that not only maintaining a low blood level of LDL-C but also an increase in the size of LDL particles are essential for the prevention of atherosclerosis. In a previous study, ezetimibe combination therapy and statin monotherapy reportedly showed similar sdLDL-C-lowering effects [22]. However, there has been no report about patients with type 2 diabetes mellitus who were already receiving statin therapy, and this is the first study to show that ezetimibe combination therapy has a greater sdLDL-C-improving effect than statin monotherapy in patients already using statins.

Oxidized LDL, including MDA-LDL, is a marker of oxidative stress, and has been reported to serve as a predictor of cardiovascular events [23]. Oxidized LDL is closely related to progression of atherosclerosis, including the formation of foam cells, vascular endothelial dysfunction, and vascular inflammation [24]. In coronary artery disease, diabetes mellitus, and metabolic syndrome, the level of oxidized LDL has been reported to be high [25], and it is thought to be closely involved in the pathology of diabetes as well as the progression of vascular complications. Regarding changes of MDA-LDL, only a few reports have compared statin monotherapy with ezetimibe combination therapy, and none of the previous studies targeted only patients with diabetes mellitus. In the present study, ezetimibe combination therapy showed a greater MDA-LDL-lowering effect than statin monotherapy, which was similar to the finding reported by Uemura et al. [26], who studied patients with impaired glucose tolerance and coronary artery disease. The effectiveness of this combination therapy in patients with diabetes mellitus was thus emphasized.

Remnant lipoproteins accumulate in the vessel wall, and are involved in the onset and progression of atherosclerosis, such as foam cell formation, vascular endothelial dysfunction, promotion of vascular smooth muscle proliferation, and production of PAI-1 [27]. Remnant lipoprotein levels rise from the stage of abnormal glucose tolerance [28], and reportedly increase even without the presence of obvious dyslipidemia [29]. In other words, in patients with diabetes mellitus, qualitative abnormalities of lipoproteins may be one of the reasons why the risk of cardiovascular events is so high. Some studies have compared statin monotherapy and ezetimibe combination therapy in patients with abnormal glucose tolerance and coronary artery disease [26,30], but there has never been a study only targeting patients with diabetes mellitus. In the present study, the percent reduction of TG and atherosclerosis-inducing RLP-C, so-called “TG-rich lipoprotein”, was significantly higher in the combined group, suggesting that combination therapy with ezetimibe can be useful for suppressing the onset and progression of atherosclerosis by controlling remnant lipoproteins in diabetic patients.

In the present study, the levels of TG, sdLDL-C, RLP-C, and MDL-LDL were markedly improved in the group receiving concomitant treatment with ezetimibe. The mechanism by which ezetimibe decreases TG levels remains unclear, although this drug has been suggested to decrease TG levels by inhibiting cholesterol absorption, chylomicron production, fatty acid absorption via FATP4, and apoB-48 production in the small intestine [31-33]. Regarding the improvement of sdLDL-C and RLP-C, ezetimibe is considered to inhibit chylomicron production and thereby reduce the TG-rich lipoproteins, which means that this drug decreases the LDL-C level and normalizes the size of LDL particles [31] and thereby decreases both sdLDL-C and RLP-C levels. Regarding the improvement of MDA-LDL, ezetimibe decreases the level of sdLDL-C, which is likely to be oxidized and degraded [34], and it inhibits the absorption of dietary oxysterol that is also likely to be oxidized and degraded [35].

It has been reported that ezetimibe improves insulin resistance in animals [36] and humans [37]. It has also been reported that ezetimibe improves insulin resistance in mice fed a high-fat diet, but not in mice fed a normal diet. This suggests that ezetimibe may normalize the high-fat diet-enhanced activity of SREBP-1c and thereby improve hepatic insulin resistance [38]. Tsunoda et al. reported that ezetimibe improved insulin resistance in their patients with high insulin resistance (mean HOMA-IR: 5.3) [37]. In the present study, 44% of the subjects were taking pioglitazone and 36% were taking metformin before the start of the study, so their insulin resistance was improved (mean HOMA-IR:2.8) at the time of the study. Thus, it is considered that the effect of ezetimibe on insulin resistance might not have been evaluated properly.

This study had the following limitations. The first limitation was a relatively small sample size, and the observation period was also short. The second limitation is that the present study used surrogate markers for cardiovascular events to examine the effectiveness of statin monotherapy and ezetimibe combination therapy. The results of the currently ongoing IMPROVE-IT study targeting patients with acute coronary syndrome will help to determine which of these therapies can actually prevent atherosclerotic disease.

## Conclusions

In hypercholesterolemic patients with type 2 diabetes, combination therapy with 2.5 mg/day of rosuvastatin and 10 mg/day of ezetimibe achieved significantly greater improvement of not only LDL-C but also sdLDL-C, MDA-LDL, TG, and RLP-C compared with dose escalation of rosuvastatin monotherapy. In addition, the results revealed that rather than simply decreasing serum lipids, combined therapy can also produce qualitative improvement of the lipid profile and shift it toward a more favorable profile for the suppression of atherosclerosis. Strong statins are not sufficiently effective for controlling lipid levels and attaining the target levels in diabetic patients. The results of the present study may suggest a useful alternative approach when statin therapy fails to control lipid levels in diabetic patients who are prone to coronary artery disease.

## Abbreviations

LDL-C: Low-density lipoprotein cholesterol; HDL-C: High-density lipoprotein cholesterol; JDCS: Japan diabetes complications study; TG: Triglycerides; sdLDL-C: Small dense low-density lipoprotein cholesterol; oxidized LDL: Oxidized low-density lipoprotein cholesterol; NPC1L1: Ezetimibe inhibits niemann-pick C1-like protein 1; NGSP: National glycohemoglobin standardization program; MDA-LDL: Malondialdehyde-modified low-density lipoprotein; RLP-C: Remnant-like particle cholesterol; HOMA-IR: Insulin resistance index; JDS: Japanese standard substance; NCEP ATP III: National cholesterol education program adult treatment panel III; MetSyn: Metabolic syndrome; ABC: ATP-binding cassette transporters.

## Competing interests

Y. Tanaka, has received consulting fees, speaking fees, and/or honoraria from Mitsubishi-Tanabe, Eisai, Chugai, Abbott Japan, Astellas, Daiichi-Sankyo, Abbvie, Janssen, Pfizer, Takeda, Astra-Zeneca, Eli Lilly Japan, GlaxoSmithKline, Quintiles, MSD, Asahi-Kasei and has received research grants from Bristol-Myers, Mitsubishi-Tanabe, Abbvie, MSD, Chugai, Astellas, Daiichi-Sankyo. Y. Okada has received consulting fees and speaking fee from Takeda Industrial Pharma, MSD, and Novartis Pharma.

## Authors’ contributions

All authors listed on the manuscript participated in the design of the study and in writing the manuscript. KT performed the statistical analysis. All authors read and approved the final manuscript.
